# On optimal pooling designs to identify rare variants through massive resequencing

**DOI:** 10.1002/gepi.20561

**Published:** 2011-01-19

**Authors:** Joon Sang Lee, Murim Choi, Xiting Yan, Richard P Lifton, Hongyu Zhao

**Affiliations:** 1 Department of Epidemiology and Public Health, Yale UniversityNew Haven, Connecticut; 2 Department of Genetics, Howard Hughes Medical Institute, Yale University School of MedicineNew Haven, Connecticut; 3 Keck Laboratory, Yale UniversityNew Haven, Connecticut

**Keywords:** optimal pooling designs, rare variant detection, next-generation sequencing

## Abstract

The advent of next-generation sequencing technologies has facilitated the detection of rare variants. Despite the significant cost reduction, sequencing cost is still high for large-scale studies. In this article, we examine DNA pooling as a cost-effective strategy for rare variant detection. We consider the optimal number of individuals in a DNA pool to detect an allele with a specific minor allele frequency (MAF) under a given coverage depth and detection threshold. We found that the optimal number of individuals in a pool is indifferent to the MAF at the same coverage depth and detection threshold. In addition, when the individual contributions to each pool are equal, the total number of individuals across different pools required in an optimal design to detect a variant with a desired power is similar at different coverage depths. When the contributions are more variable, more individuals tend to be needed for higher coverage depths. Our study provides general guidelines on using DNA pooling for more cost-effective identifications of rare variants. *Genet. Epidemiol*. 35:139-147, 2011. © 2011 Wiley-Liss, Inc.

## INTRODUCTION

Genome-wide association studies (GWAS) have enjoyed a great success in the past several years to localize disease-susceptibility loci for many common traits and diseases. The current GWAS paradigm was partially motivated by the common disease common variant (CDCV) assumption, which postulates that a large proportion of heritability of common diseases is due to common variants. GWAS was made possible by both technological advances that can type hundreds of thousands of single nucleotide polymorphisms (SNPs), at affordable cost and the strong dependency, called linkage disequilibrium (LD), among SNPs at the population level. The presence of LD allows researchers to capture the genetic variations in a person's genome by a set of tagSNPs which can be selected based on the LD patterns to factor associations between diseases and disease-causing loci indirectly. One key to the success in GWAS lies in how strong the correlations between tagSNPs and disease-causing loci are. From this CDCV perspective, GWAS have been successful in uncovering many common SNPs associated with common diseases including type I/II diabetes, rheumatoid arthritis, Crohn's disease, and coronary heart disease. However, as noted in Hardy and Singleton [2009], the combination of many identified common variants only explains a small proportion of the genetic component of the common diseases. One possible explanation of this limitation is that GWAS have focused on variants that are common (minor allele frequencies >5%), whereas many disease-causing variants are rare and therefore difficult to be tagged by common variants.

Recently, researchers have explored the possibility of an alternative hypothesis, the common disease rare variant assumption, which states that the diseases are caused by combinations of multiple rare genetic variants. Gorlov et al. [2008] found that the minor allele frequency (MAF) distribution of possibly and probably damaging SNPs is shifted toward rare SNPs compared with the MAF distribution of benign and synonymous SNPs based on the prediction results obtained from PolyPhen. Li and Leal [2008] pointed out that multiple rare variants have been implicitly identified to be associated with diseases such as obesity and schizophrenia. Low frequencies of rare variants lead to weak correlations with tagSNPs. As a result, GWAS are low-powered to detect rare variants. Consequently, different approaches are required for the detection of rare variants. At present, sequencing of candidate genes or entire genomes seems to be a good strategy to identify rare variants as claimed in Li and Leal [2009].

Next-generation sequencing (NGS) or massively parallel sequencing technologies (454FLX, Illumina/Solexa Genome Analyzer, ABI SOLiD. See Mardis [2008] for a review) have brought immense evolution in biological research and increased our biological knowledge underlying diseases. New sequencing technologies have enabled the process of millions of sequence reads of short lengths (35–250 bp, depending on the platform) at a time. Only one or two instrument runs may be required to complete a sequencing experiment. This technological breakthrough has given rise to an international research consortium, 1,000 Genomes Project (1,000GP), where the scientists will sequence the genomes of at least 1,000 people from different ethnic groups.

NGS technologies have opened up great opportunities for discovering more variants in the human genome. Whole exome sequencing technology is emerging as an effective way of capturing a patient's functional rare variants. However, whole genome or exome sequencing cost is still high although researchers are endeavoring to bring down the cost of sequencing a whole genome as low as $1,000 [Service, 2006]. In addition, thousands of genomes need to be sequenced in order to find rare SNPs with MAFs ∼1%. Consequently, a cost-effective procedure is needed to most efficiently employ the NGS methods to identify rare variants. The issues on a practical limit of cost and labor could be resolved by the use of pooling the genomic DNAs from a relatively low number of individuals. DNA pooling has been used to reduce the cost of large-scale association studies based on high-throughput genotyping technologies. [For reviews see Norton et al., 2004; Sham et al., 2002.] For GWAS, the use of DNA pooling has been considered as a cost-efficient initial screening tool to detect candidate regions in a two-stage design. In the first stage, a case-control association test for each marker is performed based on the estimated allele frequencies from the case and control pools. In the second stage, the candidate markers selected from the first stage are re-evaluated by individual genotyping [Zhao and Wang, 2009; Zou and Zhao, 2004; Zuo et al., 2006]. As suggested by Out et al. [2009], the use of a pooled DNA sample for targeted NGS also can be an attractive cost-effective method to identify rare variants in candidate genes. In their paper, a Poisson model was employed to calculate the mis-detection probability and similarly the power to detect a variant. In the calculation of the mis-detection probability, they did not take into account the dependency among incorrect bases. Moreover, the proposed statistical power represents the probability of identifying a variant present in a given pooled sample so that the probability of including the variant in the sample is not included in the power calculation. However, it is very important to reflect the sampling variation in the power calculation for pooling designs. In this paper, considering both issues, we investigate the detection probability of a variant in DNA pooling for NGS, and the optimal pooling designs.

This paper is organized as follows. In the next section, we will describe how to estimate the detection probability of a variant with a MAF *p* at a coverage depth *C* in a DNA pool of *k* individuals. Due to technical variations in DNA pooling and exon capturing, the contribution of each individual may not be equal. Therefore, we will discuss how to evaluate the average detection probability allowing individual variations in the pooled DNA sample. We illustrate these points with a real sequencing data set in the subsequent section. We conclude this paper with some technical details discussed in Appendix A.

## METHODS

Suppose that a pooled DNA sample *j* is constituted by combining DNA from *k* individuals. Let 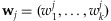 denote the proportional contributions of the *k* individuals in the *j*th pooled DNA sample to be analyzed by a NGS platform. Therefore, 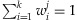 and each 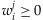. We assume that 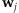 is invariant to genome positions. As detailed later, from the comparison between genotyping results and sequencing results from an empirical study, the contribution of each individual to resulting base reads can be estimated as shown in Appendix A.2 and empirical data suggest that the variations can be substantial across individuals. Moreover, in a practical pooling study, those contributions are often unknown. The objective of this paper is to assess how likely a variant with a MAF *p* can be detected from a pool of *k* individuals when the sequencing coverage depth at the position is *C*. As shown later in this section, **w**_*j*_ is a key component in the calculation of the detection probability of a variant. In the following discussion, we call a variant detected if at least *T* sequence reads carry this variant.

First, we begin with the assessment of the detection probability of a rare variant in the simplest setup. In this case, the contribution is assumed to be equal across the individuals in a pooled sample. Suppose that there is a total of 2*k* chromosomes among the *k* individuals. Let *N* denote the number of chromosomes among them carrying the rare variant. Then the detection probability of a variant with a MAF *p* can be calculated as follows:

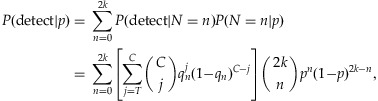
1where *n* is the number of chromosomes carrying the variant in a sample, *T* is the threshold to call the presence of the minor allele in the sample, *C* is the coverage depth, and 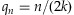 for 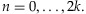




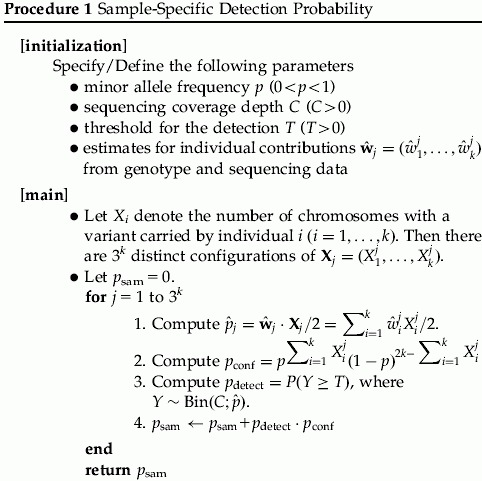


Generally, for each sample *j*, the individual contributions may not be equal, that is, the **w**_*j*_ may differ. From this perspective, it is desirable to evaluate the detection probability under a specific distribution for **w** when it varies. The randomness of the individual contributions in a pooled sample can be represented by the specification of a prior distribution for **w**. A natural choice for this distribution of **w** is the *Dirichlet* distribution with hyperparameters 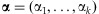, where 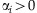 for 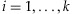. Due to exchangeability among the sampled individuals, we may assume 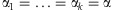. For the hyperparameters **α**, we may either specify a hyperprior distribution or estimate **α** empirically. For a specific **α**, we can use Procedure 1 to estimate the detection probability. To get a sense of the α value in practice, we have gathered empirical data to estimate α. In Appendix A, we describe the empirical data and the estimation procedure. We will call this estimator 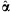 as the pseudo maximum-likelihood estimator (PMLE) or the pseudo method of moments estimator (PMME). See Appendix A.3 for more details. We illustrate how to compute the average detection probability in Procedure 2.



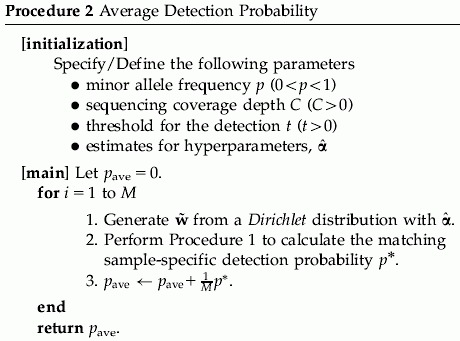


Up to this point, the estimation of the detection probability is based on the use of a single lane. However, if *L* lanes are used to analyze independent samples, then the detection probability can be computed as follows:

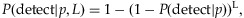
2where *P*(detect|*p*) is calculated by Equation (1) or Procedure 2.

## RESULTS

### EQUAL CONTRIBUTIONS

**Figure 1:**
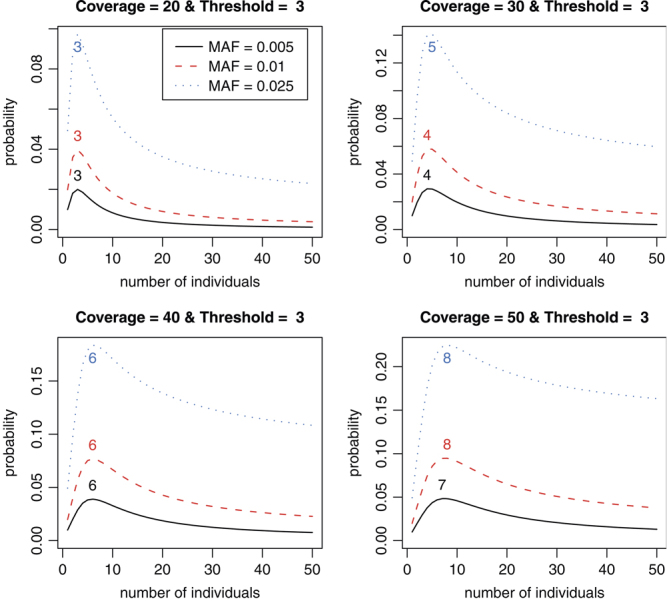
The optimal numbers of individuals on detection probabilities of the variants of *P* = 0.005, 0.01, and 0.025 with *C* = 20, 30, 40, and 50, threshold *T* = 3, and equal contributions. The number on each curve is the optimal number of individuals.

We calculate the detection probability for a given number of individuals in a pooled sample for a given coverage depth, threshold, and MAF. Therefore, the optimal number of individuals in a pooled sample can be determined in terms of the detection probability. In addition, we study the number of lanes required to reach a certain level of statistical power to identify a rare variant. Since our interest lies in the identification of a rare variant, we choose 0.005, 0.01, and 0.025 for MAFs in our analysis. We use several coverage depths *C* = 20, 30, 40, and 50 and a fixed threshold, *T* = 3. The choice of a threshold *T* is discussed in more details in the Discussion and Appendix sections. As shown in Figure 1, the detection probability initially increases with more individuals in a pool but then decreases from a certain point. This phenomenon can be explained by using Equation (1). We focus on rare variants in this manuscript, and only a small number of chromosomes among 2*k* chromosomes tend to carry the variant in a given pooled sample for such variants even when the pool size *k* increases. For example, consider a variant of a MAF equal to 0.01 and the pool size 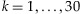. The probability that the number of chromosomes carrying the variant is at most 2 is above 97% for 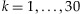. Therefore, if pools have the rare variant, most of the pools will have the variant on 1 or 2 chromosomes among the 2*k* chromosomes. In addition, it is more likely that only one chromosome holds the variant in those pools. As a result, Equation (1) may be approximated by 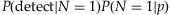. As the pool size *k* increases, the sampling probability *P*(*N* = 1|*p*) increases (due to the presence of more chromosomes), whereas the conditional detection probability *P*(detect|*N* = 1) decreases (due to the threshold set to declare the presence of a rare variant). These two factors counter balance each other and lead to an optimal number of samples in a pool. For example, if the pool size *k* increases from 3 to 30, the probability that only one chromosome carries the variant among 2*k* chromosomes increases about eightfold from 0.03 and 0.22. However, when the coverage is *C* = 20 and the threshold is *T* = 3, the detection probability conditional on having only one chromosome harboring the variant decreases much more significantly from 0.67 to 0.004 since the proportion of the chromosomes in the pool carrying the variant drops from 1/6 to 1/60, making it more difficult to detect the rare variant. It is also interesting to note that the optimal numbers of individuals in a pooled sample is somewhat invariant to MAFs. However, significantly more lanes and subsequently more individuals are required to detect a variant of a lower MAF to achieve a certain level of statistical power. Table I also shows that the total number of individuals is about the same across different options for the same MAF.

**Table I:** The optimal numbers of individuals per lane for a given coverage depth based on the uniform individual contributions

*C*	*p*	Indv	Prob	Lane	Total	*C*	*p*	Indv	Prob	Lane	Total
	0.005	3	0.0200	80	240		0.005	4	0.0293	55	220
20	0.010	3	0.0397	40	120	30	0.010	4	0.0580	27	108
	0.025	3	0.0973	16	48		0.025	5	0.1408	11	55
	0.005	6	0.0390	41	246		0.005	7	0.0482	33	231
40	0.010	6	0.0768	21	126	50	0.010	8	0.0947	17	136
	0.025	6	0.1836	8	48		0.025	8	0.2247	7	56

Indv, the optimal number of individuals; Prob, detection probability; Lane, the minimum number of lanes required for 80% power; Total, the total number of individuals required for 80% power.

### UNEQUAL CONTRIBUTIONS

For the unequal contribution case, we first need to consider the distribution of the unknown contribution **w**_*j*_ for a given sample *j*. The estimation of **w** from sequencing and genotyping information is described in Appendix A.2. The proposed estimation approach for **w** was applied to a sample data (For the data description, see Appendix A.1.), and our estimate 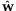 is (0.1380, 0.0836, 0.1142, 0.0188, 0.1805, 0.1364, 0.1617, and 0.1667). It is apparent that there were less contributions of individuals 2 and 4 to the pool.

**Figure 2:**
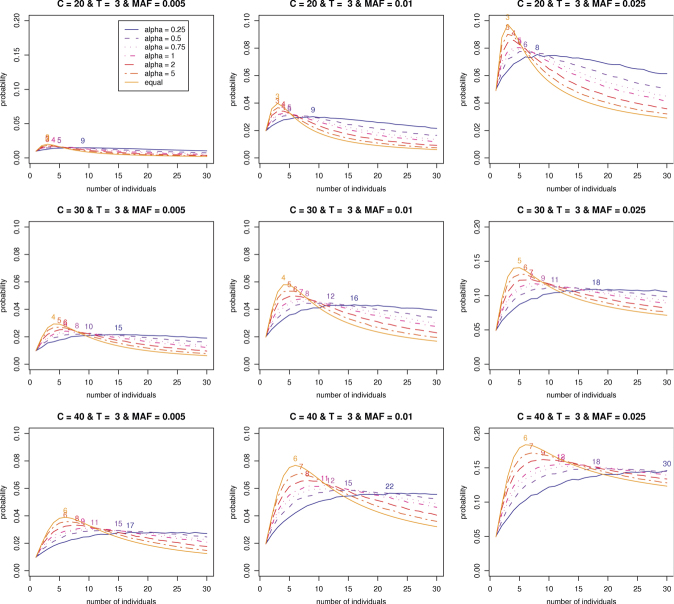
The optimal numbers of individuals on average detection probabilities of variants of *P* = 0.005, 0.01, and 0.025 with coverage depths *C* = 20, 30, and 40 and threshold *T* = 3, and the Hyperparameter α = 0.25, 0.5, 0.75, 1,2, and 5. The number on each curve is the optimal number of individuals.

Assuming that the **w** are drawn from a *Dirichlet* distribution, we first explore the effect of α on the optimal number of individuals in a pooled sample. We select a set of different values for the hyperparameter, α = 0.25, 0.5, 0.75, 1.2, and 5. We know that the variability for each individual contribution decreases with an increase in the hyperparameter α in the *Dirichlet* distribution. In this sense, **w** should be generated more closely around the mean 1/*k* for larger α. Consequently, it can be seen in Figure 2 that as α increases, the matching optimal number of individuals gets smaller and closer to the one based on the equal contributions. It can be also found that the average detection probability increases with the magnitude of α.

**Figure 3:**
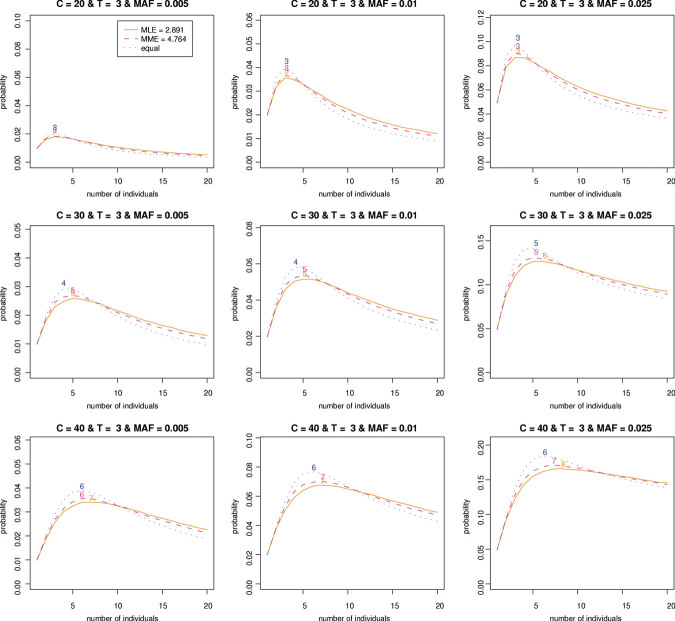
The optimal numbers of individuals on average detection probabilities of variants of *P* = 0.005, 0.01, and 0.025 with coverage depths *C* = 20, 30, and 40 and threshold *T* = 3. The hyperparameters 2.89 and 4.76 are estimated by the PMLE and PMME, respectively. The number on each curve is the optimal number of individuals. PMLE, pseudo maximum-likelihood estimator; PMME, pseudo method of moments estimator.

Now, we are interested in the estimation of the hyperparameter α based on 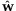
from our empirical data. As described in Appendix A.3, we can estimate the hyperparameter α by PMLE or PMME. The estimates are 2.89 and 4.76, respectively, based on PMLE and PMME. By utilizing these two estimates of α, average detection probabilities are calculated with various sequence read coverage depths *C* = 20, 30, 40, and 50 and MAFs *P* = 0.005, 0.01, and 0.025. Like the equal contribution case, we investigate how many individuals and/or lanes should be used to best identify a rare variant with a fixed coverage depth based on the given estimates. From Figure 3 and Table II, we can find patterns similar to the one for the equal contribution case. However, the results show that more individuals per lane and more lanes are required in order to obtain a given level of statistical power compared to the equal contribution case. In addition, the resulting detection probabilities are shown to decrease 7–10% in comparison with the ones for the equal contribution case (Tables I and II).

**Table II:** The optimal numbers of individuals per lane for a given coverage depth based on MLE and MME

MLE (α = 2.89)						MME (α = 4.76)											
*C*	*p*	Indv	Prob	Lane	Total	*C*	*p*	Indv	Prob	Lane	Total
	0.005	3	0.0180	89	267		0.005	3	0.0183	89	267
20	0.010	3	0.0357	45	135	20	0.010	3	0.0368	43	129
	0.025	3	0.0868	18	54		0.025	3	0.0904	17	51
	0.005	5	0.0259	62	310		0.005	5	0.0268	60	300
30	0.010	5	0.0515	31	155	30	0.010	5	0.0539	30	150
	0.025	6	0.1265	12	72		0.025	5	0.1307	12	60
	0.005	7	0.0340	47	329		0.005	6	0.0356	45	270
40	0.010	7	0.0676	23	161	40	0.010	7	0.0703	23	161
	0.025	8	0.1661	9	72		0.025	7	0.1710	9	63
	0.005	10	0.0423	38	380		0.005	9	0.0442	36	324
50	0.010	10	0.0838	19	190	50	0.010	8	0.0872	18	144
	0.025	12	0.2046	8	96		0.025	11	0.2100	7	77

Indv, the optimal number of individuals; Prob, detection probability; Lane, the minimum number of lanes required for 80% power; Total, the total number of individuals required for 80% power. MME, method of moments estimator; MLE, maximum-likelihood estimator.

## DISCUSSION AND CONCLUSION

In this paper, we have considered the detection probability of a variant when a NGS platform is utilized to identify a rare variant through DNA pooling. Through the use of an empirical data set, we inspected the number of lanes and individuals per lane needed to be able to locate a rare variant with a given chance. In this examination, a number of interesting properties are uncovered. First, increasing the number of individuals makes the detection probability higher initially up to a certain point and afterward the detection probability decreases with the number of individuals. Therefore, we can determine the optimal number of individuals in a single lane for a given MAF, coverage depth, and threshold. Second, the optimal number of individuals per lane is very close across MAFs but many more lanes are needed for the identification of a rarer variant at a given level of detection probability. For a higher coverage depth *C* and MAF *p*, the optimal number of individuals increases.

As introduced at the beginning of this article, Out et al. [2009] also carried out the analysis of detecting rare variants. We found that there are a number of differences in their analysis compared to our approach. First, they defined the mis-detection probability by

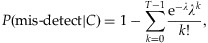
3

where λ is the mis-sequencing rate, that is, λ = *C*·*p* when *C* and *p* denote the local coverage depth and sequencing error rate, respectively. Due to sequencing errors, it is possible to have up to three incorrect minor alleles among which there are the dependency. Unlike the calculation of the mis-detection probability shown in Appendix A.4, Equation (3) cannot take the dependency into account. Second, they focused on the identification of a variant present in a given pooled sample in their power analysis. However, when we collect samples for DNA pooling, we cannot guarantee that those samples include a specific variant. In this sense, it is crucial to consider the sampling variation in the power calculation as can be seen in Equation (1). Last but not the least, the power calculation in our work can take into account the variations of individual samples in a pooled sample by making the use of results from microarray-based genotyping and NGS DNA sequencing, whereas their power calculation is based on the assumption that individual contributions are equal.

In our analysis, a very simple model is employed without taking into account the variations such as sequencing errors. Sequencing errors were estimated to be between 1 and 3% [Illumina, 2009; Richter et al., 2008]. The sequencing error rates are currently expected to be between 0.5 and 1% due to the advance in sequencing technologies. This level of sequencing errors will add very little effects to the results on detection probabilities discussed above.

**Figure 4:**
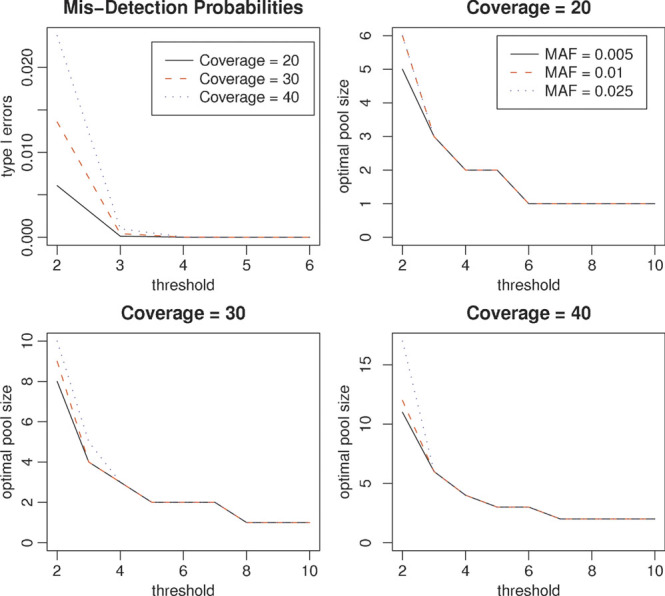
The mis-detection probabilities against thresholds (upper left) and optimal pool sizes for variants of MAFs *P* = 0.005, 0.01, and 0.025 with the coverage depth *C* = 20, 30, and 40. MAF, minor allele frequency.

In this article, we have considered a threshold of 3 for the detection of a rare variant. This is a somewhat conservative threshold as the probability that a non-existent variant is detected three times or more at the discussed coverage depth is very small at an overall sequencing error rate of 0.5–1% if the errors were to occur independent of each other. We choose to err on the conservative side due to potential non-independence of the sequencing errors and the large number of bases investigated. At an overall sequencing error rate of 1% and a coverage depth of *C* = 20, 30, 40, and 50, assuming a base has an equal chance to be mis-sequenced to one of the three other bases, the chance that an incorrect base is observed twice (three times or four times) or more is shown in Table IV and Figure 4. The results show that the use of threshold of 3 controls those mis-detection probabilities at the level of 0.2% across given coverage depths of *C* = 20, 30, 40, and 50. In order to control the mis-detection probability more stringently, a larger threshold than *T* = 3 may be preferred. As shown in Figure 4, the optimal numbers of individuals in a pooled sample is still similar across different MAFs for given coverage depth and larger threshold. These results also suggest that controlling the mis-detection probability more stringently requires the use of a smaller pooled size. Additionally, in Appendix A.5, we also briefly describe how to construct a random threshold for yielding an exact mis-detection probability for a given significance level and perform our analysis. See Appendix A for more details. To summarize, our study has shown that DNA pooling can be a very cost-effective approach for detecting rare variants, and the optimal number of individuals in a pool is robust to the MAFs of rare variants at a specific coverage depth. This is a very desired property as the rare variants to be discovered have unknown frequencies. Moreover, DNA pooling can also be a very effective approach for genetic association studies, and this will be explored in our future work.

## APPENDIX A A.1. DATA DESCRIPTION

For our analysis, we use an empirical data set from a pooled sample consisting of the genomic DNAs from eight individuals. Exome DNA sequencing was performed by first capturing the exome using NimbleGen 2.1M human exome array, followed by Illumina genome analyzer sequencing. The resulting reads are 75 bp paired-end reads. For the alignment and mapping of sequencing reads, we used Bowtie. The genomic DNAs from the eight individuals were also genotyped on Illumina 610-Quad (San Diego, CA) SNP array. As a result, both base counts and genotypes are available for 11,312 positions in the exome.

## A.2. ESTIMATION OF THE CONTRIBUTIONS OF INDIVIDUALS IN A POOLED SAMPLE

A common approach to evaluating the accuracy of NGS SNP detection is to compare the sequencing results with those from microarray-based genotyping platforms. Microarray-based genotyping information can be also utilized in the assessment of the rate of discovery for a variant from a NGS technology. If there is genotyping information available for all the individuals in the pooled DNA sample *j*, the individual contributions in the sample can be estimated in the following way. Let  denote the proportion of the major allele at a DNA position *l*  for the *i*th individual so that . Then

A.1

where , , and  is the expected major allele frequency of the position *l* acquired by sequencing the given pooled sample *j* . Based on results from microarray-based genotyping and DNA sequencing, we can formulate a quadratic programming optimization as follows:

A.2

where  is the *l*^2^ norm,  is the *i*-th row of **X**, and  is the vector of sample major allele frequencies from the NGS method. Note that we impose the constraint to ensure the non-negativity of the individual contributions to the overall pool.

## A.3. ESTIMATION OF THE HYPERPARAMETER FOR A DIRICHLET DISTRIBUTION

The (pseudo) maximum-likelihood estimate (afterward MLE)  cannot be expressed in closed-form but can be obtained by making the use of an iterative scheme such as *Newton-Raphson* method [Minka, 2009]. Here, we briefly describe *Newton-Raphson* procedure for the MLE ; The probability density for the *Dirichlet* distribution with parameters  at  is

A.3

where*w*_*i*_>0 for each  and . If  were available, then the log-likelihood could be written as follows:

Note that for unknown ,  will be replaced by  in this step. The first and second derivatives of the log-likelihood are given by

and

respectively, where . The Newton-Raphson method would be performed iteratively by

until  for a precision ⋯.

Our second estimator is based on the method of moments. Under the assumption , for each ,

so that

A.4

The MME (PMME) can be determined by replacing *s*^2^ by the sample variance of all *w*'s ( 's).

For the comparison of the above two estimators, we performed a simulation with α = 2, *k* = 8, and 20,000 iterations. As shown in Table III, every pair in those summary statistics are very comparable even though the median and mean for the MME are closer to the true α than those for the MLE. We also conducted simulations with different α and *k* and found similar patterns.

**Table III:** Summary statistics for 20,000 MMEs and MLEs for α = 2 and *k* = 8

	MME	MLE
1st Qu.	1.3553	1.5934
Median	1.9821	2.1923
Mean	2.4394	2.7312
3rd Qu.	2.9359	3.1932
Bias	0.4394	0.7312
Var	3.6335	4.2745

MME, method of moments estimator; MLE, maximum-likelihood estimator.

## A.4. MIS-DETECTION PROBABILITIES DUE TO SEQUENCING ERRORS

In this section, we describe the calculation of mis-detection probabilities due to sequencing errors. Suppose that the overall sequencing error is 1%. Let *X*_1_ and *X*_*i*_  denote the number of a true base and the number of one of three incorrect bases at a coverage depth *C*, respectively. Under the assumption that a base has an equal chance to be mis-sequenced to one of the three incorrect bases,  follows a multinomial distribution with *p*_1_ = 0.99 and *p*_2_ = *p*_3_ = *p*_4_ = 1/300. Note that

where . Then the mis-detection probability for a threshold of 2 is calculated as follows:

where *C* is a coverage depth. We can also compute the mis-detection probability for *T*≥3 in a similar way. The mis-detection probabilities for *T* = 2, 3, and 4 can be found in Table IV.

**Table IV:** The probabilities that an incorrect base is observed twice (three times/four times) or more with a coverage depth of *C* = 20, 30, 40, and 50 based on the overall sequencing error rate of 1%

	*C* = 20	*C* = 30	*C* = 40	*C* = 50
≥2	0.006075	0.013573	0.023730	0.036307
≥3	0.000121	0.000422	0.001001	0.001936
≥4	0.000002	0.000009	0.000031	0.000075

## A.5. RANDOM THRESHOLDS FOR THE MIS-DETECTION PROBABILITY OF 0.01%

Based on Table IV, we construct a random threshold for controlling type I errors of 0.01% as follows: For ,

A.5

where *t*_1_ = 3, *t*_2_ = 4, and *w* are so chosen as to satisfy the corresponding type I error is equal to 0.01% at a coverage depth *C*. See Tables V and VI for more details.

**Table V:** The specification of random thresholds for a coverage depth of *C* = 20, 30, 40, and 50 controlling the type I error rate 0.01%

	*C* = 20	*C* = 30	*C* = 40	*C* = 50
*w*	0.8235	0.2203	0.0711	0.0134
E(*T*)	3.1765	3.7797	3.9289	3.9866

**Table VI:** The optimal number of individuals per lane for given coverage depth based on the uniform individual contributions

*C*	*p*	Indv	Prob	Lane	Total	*C*	*p*	Indv	Prob	Lane	Total
	0.005	3	0.0188	85	255		0.005	3	0.0235	68	204
20	0.010	3	0.0373	43	129	30	0.010	3	0.0466	34	102
	0.025	3	0.0917	17	51		0.025	3	0.1132	14	42
	0.005	4	0.0302	53	212		0.005	5	0.0370	43	215
40	0.010	4	0.0596	27	108	50	0.010	5	0.0729	22	110
	0.025	4	0.1437	11	44		0.025	5	0.1741	9	45

Indv, the optimal number of individuals; Prob, probability; Lane, the minimum number of lanes required for 80% power; Total, the total number of individuals required for 80% power.
